# Phytase Overdoses Enhance Thermoregulatory Processes via Convection and Radiation in Japanese Quails *(Coturnix japonica)* Raised in Hot Environments

**DOI:** 10.3390/ani15172518

**Published:** 2025-08-27

**Authors:** Luiz Arthur dos Anjos Lima, Maria Isabelly Leite Maia, Delfino Isac Belarmino Afo, Amana Fernandes Maia, Fernando Guilherme Perazzo Costa, Patrícia Emília Naves Givisiez, Ricardo Romão Guerra, Camila Urbano Braz, Edilson Paes Saraiva

**Affiliations:** 1 Research Group in Bioclimatology, Ethology and Animal Welfare (BioEt), Department of Animal Science, Federal University of Paraíba, Areia 58397-000, Paraíba, Brazil; luisarthur_@hotmail.com (L.A.d.A.L.); isabelymaia12@gmail.com (M.I.L.M.); delfinoisac.afo@gmail.com (D.I.B.A.); 2 Center for Animal and Veterinary Science, University of Trás-os-Montes and Alto Douro (UTAD), 5000-801 Vila Real, Portugal; amanamedvet@gmail.com; 3 Department of Animal Sciences, Federal University of Paraiba, CCA, Areia 58397-000, Paraiba, Brazil; perazzo63@gmail.com (F.G.P.C.); patriciagivisiez@gmail.com (P.E.N.G.); 4 Department of Veterinary Sciences, Federal University of Paraiba, CCA, Areia 58397-000, Paraiba, Brazil; ricardo@cca.ufpb.br; 5 Department of Animal Sciences, University of Illinois at Urbana-Champaign, Urbana, IL 61801, USA; cbraz@illinois.edu

**Keywords:** coturniculture, heat loss, sensitive loss, thermal balance, thermal imaging, welfare

## Abstract

This study aimed to evaluate the effects of phytase enzyme supplementation on the thermoregulatory responses of Japanese quails (*Coturnix japonica*) exposed to different thermal environments. A total of 720 one-day-old laying quails were assigned to a completely randomized design with five dietary treatments (0, 500, 1000, 1500, and 3000 FTU of phytase) and three thermal conditions: thermal comfort (24 °C) and heat stress environments (30 °C and 36 °C). Each treatment had six replicates with eight quails per experimental unit. Data were collected during the early laying phase, peak egg production, and the final laying phase. Measurements included rectal and surface temperatures (assessed via thermographic imaging), allowing the calculation of core-to-surface and surface-to-environment thermal gradients. Quails exposed to severe heat stress (36 °C) showed increased heat dissipation via convection (*p* = 0.001) and radiation (*p* = 0.029) when supplemented with phytase doses above 1500 FTU/kg. Additionally, high-dose phytase supplementation reduced the cloacal temperature and optimized thermal gradients, indicating a potential protective effect of exogenous phytase in alleviating heat stress. Overall, these findings highlight phytase supplementation as a promising nutritional strategy to enhance heat tolerance, mitigate thermal stress, and improve the welfare and physiological resilience of quails throughout the production cycle.

## Simple Summary

The stress caused by rising global temperatures in recent years is one of the main challenges in poultry farming. When exposed to hot environments, quails undergo physiological changes that negatively affect their health, welfare, and productivity. Under these conditions, birds must activate mechanisms to dissipate excess heat, such as increasing heat exchange through convection and radiation, which can compromise productive performance. In recent years, nutritional strategies have been studied as alternatives to help birds cope with the harmful effects of stressful environments. One such strategy is the use of feed additives, such as enzymes, which, in addition to improving nutrient digestibility, may also positively influence the body’s thermoregulation. Phytase has gained prominence not only for its role in releasing phosphorus from diets but also for its potential effects on the physiology of birds under heat stress. As a result, nutrition studies have played a fundamental role in improving breeding systems and maintaining bird productivity in challenging environments, contributing to more sustainable poultry farming adapted to climate change.

## 1. Introduction

Global surface temperatures have been rising in recent years, and climate control systems for poultry farms remain costly. Although genetic selection has advanced industrial poultry production, emphasizing traits such as rapid growth and thermotolerance, little is known about how quails, particularly those bred for egg production, respond to thermal stress. While thermotolerance has been studied in broilers and laying hens, thermal responses to heat stress remain poorly characterized in Japanese quails, especially regarding their heat dissipation mechanisms. This knowledge gap limits the development of nutritional strategies tailored to the thermoregulatory physiology of this species. Given the ongoing advancements in poultry farming and the steady increase in ambient temperatures, the development of cost-effective strategies to enhance thermotolerance in quails raised in warm climates is essential [1].

Nutrition plays a crucial role in the well-being and performance of quails, as it is responsible for meeting their physiological and behavioral needs. However, under high-temperature conditions, feeding can negatively affect the performance of laying birds, especially when these animals are reared in confined systems. The main source of protein used in animal feed is soybean, and in addition to the agricultural industry facing sustainability challenges in recent years, soybeans also contain anti-nutritional factors, such as phytate, which limit the availability of nutrients to birds [2]. To address this, the poultry industry has introduced nutritional additives into diets with the aim of modulating adaptive responses, mainly by aiding digestion [3]. Among these, exogenous enzymes such as phytase have gained attention for improving nutrient utilization and potentially influencing physiological processes that may be linked to thermoregulation in birds, especially under heat stress conditions, although their direct effects on thermoregulation remain to be fully elucidated.

The use of exogenous enzymes, such as phytase, is widespread in poultry diets to enhance digestion and nutrient absorption. This process increases the feed efficiency of the diet and significantly influences heat production and dissipation, thus contributing to the maintenance of thermal homeostasis [4,5,6]. Recent studies suggest that using phytase at doses higher than current industry recommendations (a strategy referred to as enzyme overdosing) can optimize the utilization of phytic phosphorus and other chelated nutrients present in plant feed ingredients [5,7,8]. However, limited research has investigated how laying quails respond to phytase supplementation under warm environmental conditions. Furthermore, no studies to date have explored the effects of phytase overdosing on the efficiency of thermal loss pathways—particularly convection and radiation—which, due to their lower energy demand compared to evaporative cooling, may represent relevant routes for thermoregulation in birds exposed to mild to moderate heat stress. Understanding this relationship could help refine feeding strategies for quails under such conditions.

Under these rearing conditions, birds depend on physiological and behavioral adjustments to maintain thermal homeostasis, as they do not possess sweat glands. Heat dissipation occurs mainly through sensible thermal loss via radiation and convection, which are physiological mechanisms that demand lower energy expenditure. In more extreme situations, heat loss occurs through respiratory evaporation. However, the efficiency of these mechanisms can be modulated by environmental factors and nutritional strategies, including the use of feed additives that affect metabolic heat production and peripheral circulation. Previous studies in various bird species have already highlighted the role of thermal dissipation mechanisms, such as radiation, convection, respiratory evaporation, and conduction, as major contributors to thermoregulation [4]. Despite this, little is known about how dietary interventions—such as enzyme supplementation—might influence the predominance or efficiency of these sensible heat loss routes, particularly in Japanese quails during peak egg production.

We hypothesize that phytase overdosing enhances the efficiency of sensible heat loss (via convection and radiation) in laying Japanese quails, potentially by reducing metabolic heat production and improving peripheral circulation. This raises a central question: How does phytase overdose affect the thermoregulation of laying Japanese quails under different ambient temperatures?

This study aimed to evaluate the impact of phytase overdose on the thermoregulatory responses of laying Japanese quails exposed to different ambient temperatures. Our findings provide new insights into the thermal challenges faced by laying Japanese quails raised under varying temperature conditions and emphasize the importance of nutritional adjustments as critical modulators of thermal physiology.

## 2. Materials and Methods

### 2.1. Animals and Experimental Design

Experimental procedures and methods were carried out with ethical approval from the Federal University of Paraiba Ethics Committee, protocol number 3695120121.

This study was conducted in the bioclimatic chambers of the Animal Bioclimatology Laboratory at the Animal Science Department of the Agricultural Sciences Center, Federal University of Paraiba, in Areia, Paraiba, Brazil. The facility comprises three thermally insulated chambers equipped with integrated microprocessor control systems to regulate temperature, humidity, ventilation, air renewal, and photoperiod.

The experiment was conducted with 720 Japanese quails in the production phase, following a completely randomized design in a 5 × 3 factorial arrangement. The factors included five phytase levels (0, 500, 1000, 1500, and 3000 FTU) and three temperature conditions (24 °C, 30 °C, and 36 °C), with six replications per treatment and eight birds per replicate. Evaluations began in the eighth week of breeding, coinciding with the onset of egg production. The experiment was divided into five production cycles, each lasting 21 days. Data were collected during three key periods: the initial egg-laying phase (cycle 1), peak production (approximately 95% egg-laying rate, cycle 3), and the period of declining laying performance (cycle 5).

At the beginning of the first evaluation cycle, one bird was randomly selected from each replicate for data collection, totaling 90 quails. This selection method was used in the remaining evaluation cycles; the birds were identified with a colored ring on their paw in each cycle. The selected birds were housed in cages equipped with nipple drinkers and feeders, with food and water provided ad libitum. Thirty birds were assigned to each of the three rooms with different thermal environments: thermoneutral (24 °C, Environment 1), moderate heat stress (30 °C, Environment 2), and severe heat stress (36 °C, Environment 3). Environmental conditions were monitored and controlled throughout the experimental period. Temperature settings were maintained based on previous studies on heat stress in poultry [9].

### 2.2. Characterization of the Environment

The thermal environments were assessed using the Globe Temperature and Humidity Index (GTHI), as proposed by Buffington et al. [10]. This index evaluates the environmental thermal conditions by integrating key climatic elements relevant to birds, including temperature, radiation, and humidity. This index provides a comprehensive measure of the thermal environment, allowing for a more accurate assessment of heat stress conditions in poultry housing. The GTHI is calculated as follows:

GTHI = 0.5 × (BGT + AT) + 0.5 × (BGT − AT) × RH

where BGT is the black globe temperature; AT is the air temperature; and RH is the relative humidity of the air.

The Enthalpy Comfort Index (ECI) is described as a key parameter for assessing the thermal comfort of animals [11,12]. This index quantifies the amount of thermal energy (kJ) contained in 1 kg of dry air, providing a more comprehensive measure of environmental comfort. The ECI is calculated using temperature, relative humidity, and local atmospheric pressure. Based on the equation proposed by Rodrigues et al. [13], the ECI is determined as follows:

h = 1.006 × t + RH/BP × 10((7.5 × t)/(237.3 + t)) × (71.28 + 0.052 × t)

where h is the enthalpy (kJ/kg dry air); t is the temperature (°C); RH is the relative humidity of the air (%); and BP is the local barometric pressure (mmHg). The barometric pressure was set at 760 mmHg.

In this study, the averages obtained for the Enthalpytic Comfort Index (ECI) and the Global Temperature and Humidity Index (GTHI) classified the birds in different thermal environments. The 24 °C environment provided the birds with favorable conditions for thermal homeostasis, with an average GTI of 72 ± 1.0 and an average ICI of 55 ± 2.0, which characterizes it as an environment within the limits of thermoneutrality. The other environments were configured as moderate thermal stress, with average values of 80 ± 1.0 for GTI and ICI of 72 ± 1.0, and severe of 89 ± 1.0 (GTI) and 94 ± 2.0 (IC).

### 2.3. Experimental Diets

The experimental diets were formulated with corn and soybean meal, according to the nutritional recommendations of Rostagno et al. [14]. The only variation among diets was the level of phytase supplementation, with adjustments to calcium (Ca) and phosphorus (P) levels based on the commercial enzyme availability matrix for 500 FTU, which contained 0.165% Ca and 0.150% P (Table 1).

**Figure 1:**
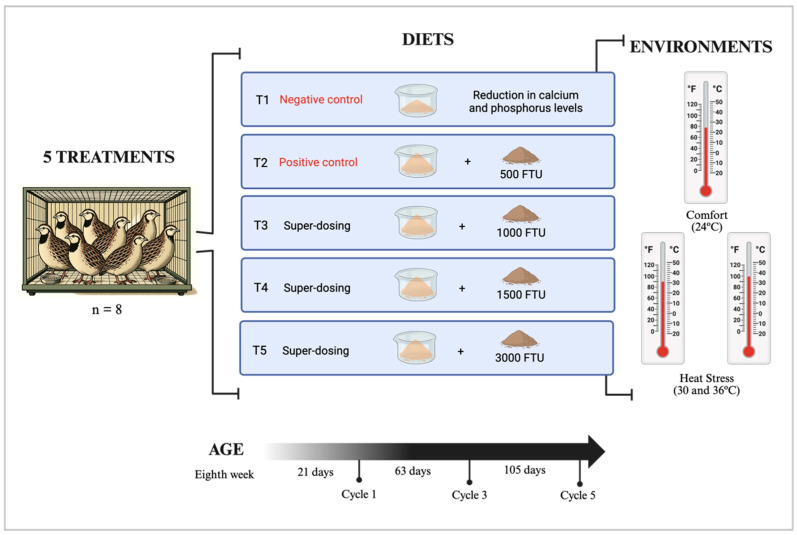
Illustration of the collection timeline and experimental diets. All birds were initially fed a basal diet and maintained under standard environmental conditions until eight weeks of age. From the eighth week onwards, birds were assigned to one of the five treatment groups and began receiving the experimental diets. One bird from each replicate was randomly selected for data collection (n = 8 per treatment in each thermal environment). Cycle 1 marked the first collection (initial laying phase), cycle 3 corresponds to the second collection (peak laying), and cycle 5 represents the final collection. Since this study was conducted in a controlled environment, the collection shift was not a factor, but all data were collected in the early afternoon (1:00 pm).

The phytase enzyme used in the diets was derived from *Escherichia coli* and produced in *Thricoderma reesei* (Quantum Blue, AB Vista, Marlborough, UK). The enzyme was incorporated into the diets by replacing part of the inert ingredient. This study included five dietary treatments: a negative control (0 FTU), with no phytase supplementation and reduced Ca and P levels; a positive control (500 FTU) balanced according to the phytase nutritional matrix recommendations for Ca and P; and three enzymes overdosing with phytase supplementation at 1000, 1500, and 3000 FTU (Figure 1).

The phytase overdose levels (1000, 1500, and 3000 FTU/kg) were selected based on previous studies that demonstrated that higher than standard doses improve nutrient digestibility, eliminate the anti-nutritional effects of phytate, and improve animal responses under stress conditions. These doses allow an exploration of the extra phosphoric effects of phytase and estimation of optimal inclusion levels through dose–response modeling, particularly relevant under heat stress conditions where thermoregulatory efficiency is critical [15].

### 2.4. Thermal Imaging

**Figure 2:**
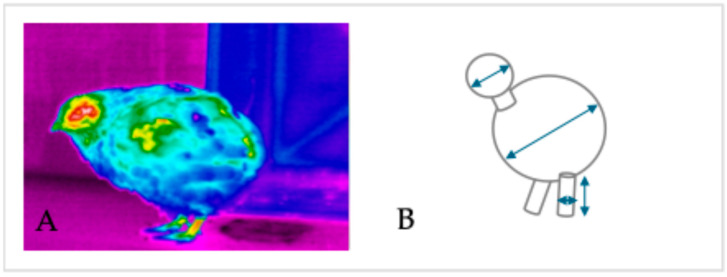
(**A**) Thermographic image of a quail in a lateral view. (**B**) Marking of the two-dimensional geometric shapes on three distinct body regions (head, back, wing, and shin) used for surface temperature measurement.

In each data collection cycle, one bird per experimental unit was randomly selected, marked with an identification ring on the paw, and transferred to a plastic floor for imaging. A Fluke thermal imaging camera (Fluke TiX500 9Hz Thermal Imager, Fluke Corporation, Everett, WA, USA, ε = 0.98; 180 × 180 pixels, accuracy ± 2%) was used to capture side-view thermal images of each bird in an upright resting position (Figure 2A). The thermographic data were then analyzed to determine the surface temperature and assess thermoregulation mechanisms, including sensible heat exchange, convection, and radiation.

### 2.5. Calculation of Heat Transfer

Thermal images were processed using Fluck Connect (version 1.1.545.0). Geometric shapes were superimposing on three distinct body regions for analysis, including the paw, back, and head (Figure 2B). Measurements of these body regions were based on the assumption that each segment corresponds to a two-dimensional shape, with the head modeled as a sphere, the back as a prolate spheroid, and the legs as open cylinders [16].

#### 2.5.1. Calculation of Convective Heat Transfer

Convective heat transfer plays a crucial role in regulating body temperature and maintaining thermal homeostasis, especially in cooling the body under hot environmental conditions. This process occurs when heat is exchanged between the animal’s body surface and the surrounding fluid (air or water) and is influenced by several factors, such as the temperature gradient between the body and the environment, the exposed surface area, the fluid properties, and the fluid movement speed [17].

Convective heat exchanges were calculated using the model proposed by McArthur [18] in the following form:

(1) $$C_{R}=\frac{\rho *C_{p}}{rh}*\left(TS-AT\right)$$

where$\;C_{R}$—convection heat exchange (W/m^2^); ρ—air density (kg/m^3^); $C_{p}$—specific heat of the air (J/kg K); *rh*—boundary layer resistance to convective heat transfer (m^2^ K/W); TS—average bird body surface temperature (K); AT—air temperature (K).

The resistance of the boundary layer to convective heat transfer was obtained using the model proposed by Frank and Nelson [19]:

(2) $$rh=\frac{\rho *C_{p}*d_{b}}{k*Nu}$$

where: *rh*—boundary layer resistance to convective heat transfer (m^2^ K/W); ρ—air density (kg/m^2^); $C_{p}$—specific heat of the air (J/kg K); $d_{b}$—average body diameter of birds (m); *k*—thermal conductivity of air (W/m K); *N_u_*—Nusselt number.

The calculation of the average diameter of birds, for each study period, was carried out using the model proposed by Mitchell [20]:

(3) $$d_{b}=0.131*p^{0.33}$$

where $d_{b}$—average body diameter of birds (cm); p—average weight of birds (g).

The Nusselt number was determined considering the representation of the birds’ bodies as spheres:

(4) $$Nu=2+0.4*R_{e}^{1/2}+R_{e}^{2/3}\;{p=P}_{r}^{0.4}$$

where *N_u_*—Nusselt number; *R_e_*—Reynolds number; *P_r_*—Prandtl number.

The Reynolds number was calculated using the following model:

(5) $$R_{e}=\frac{VV*d_{t}}{v}$$

where *R_e_*—Reynolds number; *VV*—average speed of air movement (m/s); *v*—kinematic viscosity of air (m^2^/s); *dt*—average body diameter of birds (m).

#### 2.5.2. Calculation of Radiative Heat Transfer

Radiative heat transfer occurs when bodies emit thermal energy as electromagnetic waves, without requiring physical contact or a material medium, and is influenced by factors such absolute temperature, surface area, emissivity, and temperature differences between bodies [17].

Thermal exchanges due to long-wave radiation were obtained using the calculation proposed by McArthur [18], as follows:

(6) $$L=\frac{\rho *C_{p}}{R_{r}}*\left(ST\;-T_{r}\right)\;\;\;\;$$

where *L*—heat exchange by radiation (W/m^2^); ρ—air density (kg/m^3^); *Cp*—specific heat of air (J/kg K); *Rr*—boundary layer resistance to radiation heat transfer (m^2^ K/W); *ST*—body surface temperature (K); *Tr*—mean radiant temperature (K).

The resistance of the boundary layer to heat transfer by radiation was calculated using the following equation:

(7) $$R_{r}=p*\;C_{p}*{\left(4*\varepsilon_{s}*\sigma *{{\bar{T}}^{3}}_{M}\right)}^{-1}$$

where $R_{r}$—boundary-layer resistance to radiative heat transfer (m^2^ K/W); ρ—air density (kg/m^3^); $C_{p}$—specific heat of air (J/kg K); $\varepsilon_{s}$—emissivity of bird feathers (0.94); σ—Stefan–Boltzmann constant (5.67*10−8 W/m^2^ K ^4^); ${\bar{T}}_{M}$—average temperature between TS and T_r_ (K).

The average radiant temperature was calculated using the equation proposed by Silva [21]:

(8) $${\bar{T}}_{r}={\left[\frac{1.053*h_{c}}{\sigma }*\left(BGT-AT\right)+{T^{4}}_{bg}\right]}^{0.25}$$

where ${\bar{T}}_{r}$—average radiant temperature (K); $h_{c}$—standard black globe convection coefficient; BGT—black globe temperature (K); AT—air temperature (K).

The standard black globe convection coefficient was calculated using the following equation:

(9) $$h_{c}=0.38*K*D*{R^{0.6}}_{e}*{P^{\frac{1}{3}}}_{r}\;hc$$

where $h_{c}$—standard black globe convection coefficient; k—thermal conductivity of air (W/m K); d—diameter of a standard black globe (0.15 m); Re—Reynolds number; Pr—Prandtl number.

The Prandtl number was calculated using the following equation:

(10) $$P_{r}=\frac{\rho *C_{p}*v}{k}$$

where *Pr*—Prandtl number; ρ—air density (kg/m^3^); *Cp*—specific heat of air (J/kg K); *v*—kinematic viscosity of air (m^2^/s); *k*—thermal conductivity of air (W/m K).

### 2.6. Physiological Response

In each cycle, after collecting thermographic images, cloacal temperature was measured using a digital clinical thermometer (Model TH150, G-tech, Accumed-Glicomed Medical Equipment Ltd., São Paulo, Brazil). The thermometer was inserted 2 cm into the bird’s cloaca, ensuring direct contact between the bulb and the rectal membrane. The measurement was recorded once the thermometer emitted a sound signal, indicating temperature stabilization.

The surface temperature values obtained from infrared thermography were used to calculate the average surface temperature (AST) based on the formula developed by Dahlke et al. [22]:

AST = (0.70 × Tback) + (0.12 × Twing) + (0.03 × Thead) + (0.15 × Tshin)

Using these data, the core-to-surface thermal gradient was calculated as the difference between the rectal temperature and surface temperature, and the surface-to-environment thermal gradient was determined as the difference between the surface temperature and the air temperature of each thermal environment.

### 2.7. Statistical Analysis

Heat exchange variables through convection and radiation, as well as surface temperature, cloacal temperature, and thermal gradients, were analyzed using analysis of variance (ANOVA) in R (version 4.2.0; R Core Team, 2019). Statistical significance was set at a *p*-value less than 0.05. Post hoc comparisons were performed using Tukey’s test to identify differences among treatment means, with adjustments for multiple comparisons using the emmeans package in R.

To evaluate the effects of dietary treatments, thermal environments, and production cycles, a factorial three-way ANOVA model was applied.

Yijkl = μ +αi + βj + γk + (αβ)ij + (αγ)ik + (βγ)jk + (αβγ)ijk + ϵijkl

where Yijkl is the observed value in the i-th level of factor α (phytase concentration), j-th level of factor β (thermal environment), and k-th level of factor γ (production cycle); μ is the overall mean; (αβ)ij is the effect of the interaction between α and β; (αγ)ik is the effect of the interaction between α and γ; (βγ)jk is the effect of the interaction between β and γ; (αβγ)ijk is the effect of the interaction between α, β and γ; and ϵijkl is the random error component.

For cases where the phytase concentration showed significance, the following regression model was applied:

Yi = β0 + β1Xi + εi

where Yi is the response variable for the i-th observation, β0 is the intercept, representing the expected value of Y when phytase is zero (baseline concentration), Xi is the phytase concentration for the ith observation, β1 is the slope, indicating the effect of a one-unit increase in phytase on the response variable, and εi is random error term for the ith observation, assumed to be normally distributed with a mean of zero and constant variance.

## 3. Results

### 3.1. Thermoregulatory Response

**Figure 3:**
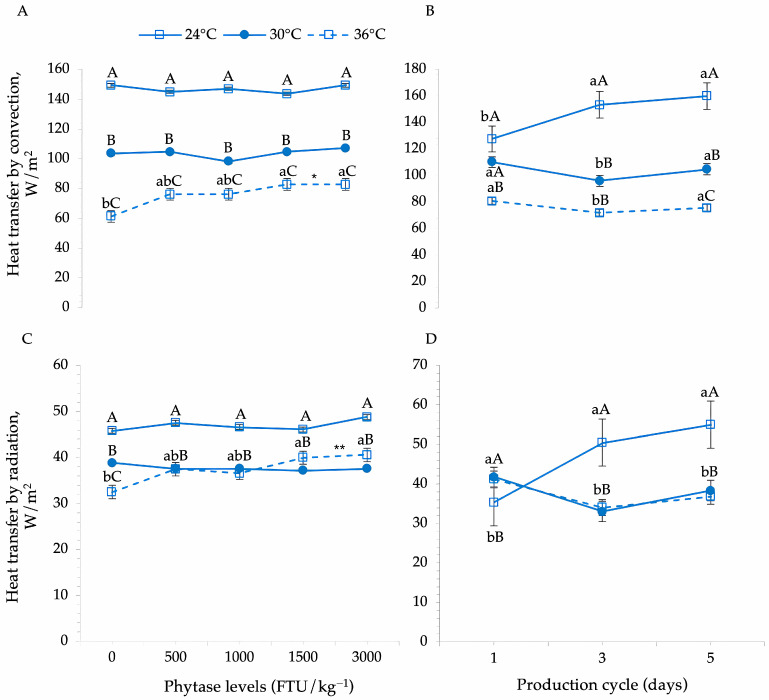
Dynamics of phytase enzyme supplementation and thermal stress on convective and radiative heat exchange (**A**,**C**), and the effects of thermal environments across production cycles (**B**,**D**) on sensible heat exchange in Japanese quails. Means followed by different lowercase letters (horizontal comparisons) and uppercase letters (vertical comparisons) differ significantly according to Tukey’s test (*p* < 0.05). * Quadratic regression equation for heat transfer by convection under severe heat stress: Y = 62.99 + 0.0201. X − 4.533 × 10^−6^. X^2^; R^2^ = 0.91; *p* = 0.014. ** Quadratic regression equation for heat transfer by radiation under severe heat stress: Y = 33.08 + 0.0062. X − 1.236 × 10^−6^. X^2^; R^2^ = 0.87; *p* < 0.05.

According to the answers related to thermoregulation (Table 2), there was a significant effect on the transfer of heat by convection (*p* = 0.001) and radiation (*p* = 0.029) of Japanese quails fed with different levels of the enzyme phytase in the different thermal environments (Table 2, Figure 3A,C).

There was an increase in convective heat transfer from 76.2 to 82.8 W/m^2^ when phytase levels increased from 1000 to 1500 FTU in the severe heat stress environment. A similar effect was observed when quails were fed diets containing 3000 FTU (Figure 3A).

The relationship between phytase levels and convective heat loss was modeled using a quadratic regression equation (Y = 62.99 + 0.0201·X − 4.533 × 10^−6^·X^2^; R^2^ = 0.91; *p* = 0.014). Quadratic regression analysis indicated a significant effect of phytase levels on convective heat exchange, exhibiting a typical plateau-shaped response curve. Inclusion of doses above 1500 FTU/kg did not result in further improvements, suggesting that this level is the optimal dose.

A similar effect was observed in radiative heat exchange, with an increase from 36.6 to 40 W/m^2^ when birds were fed diets containing 1000 to 1500 FTU. According to the quadratic regression equation (Y = 33.08 + 0.0062·X − 1.236 × 10^−6^·X^2^; R^2^ = 0.87; *p* < 0.05), doses between 1500 and 2500 FTU/kg appear to be the most biologically efficient range to enhance radiative heat exchange in Japanese quails under severe heat stress conditions (Figure 3C).

Heat transfer through convective and radiative mechanisms was also influenced by the thermal environments during the evaluated production cycles: early laying (cycle 1), peak laying (cycle 3), and post-peak laying (cycle 5) (Table 2; Figure 3B,D).

An increase in convective heat exchange was observed between the first and third production cycles in the thermal comfort environment. However, no significant differences were found between cycles 3 and 5 in this environment (Figure 3B). In moderate heat stress conditions (30 °C), convective heat exchange was highest during cycle 1 (110.1 W/m^2^), dropped to 96 W/m^2^ in cycle 3, and showed a tendency to increase again during the final cycle. A similar pattern was seen under severe heat stress (36 °C), with values decreasing from 80 W/m^2^ (cycle 1) to 71.9 W/m^2^ (cycle 3), then increasing to 75.5 W/m^2^ during the final phase.

In the thermal comfort environment, radiative heat exchange increased as the production cycle progressed. However, the opposite trend was observed in environments of moderate and severe heat stress, where radiative heat exchange declined between the beginning and peak of laying. After peak laying, radiative heat transfer increased again during the final evaluation phase (cycle 5) in these environments (Figure 3D).

### 3.2. Physiological Response

**Figure 4:**
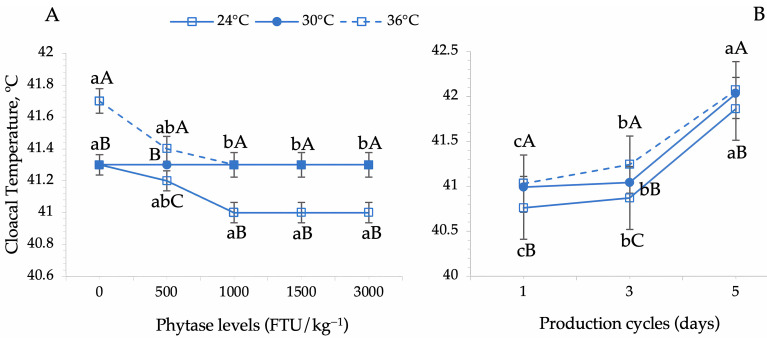
Dynamics of the cloacal temperature in Japanese quails in response to phytase enzyme supplementation and thermal environment conditions. (**A**) Effects of phytase supplementation across different thermal environments. (**B**) Cloacal temperature variations across production cycles under different environmental temperatures. Means followed by different lowercase letters (horizontal comparisons) and uppercase letters (vertical comparisons) differ significantly according to Tukey’s test (*p* < 0.05).

Cloacal temperature was significantly influenced by phytase inclusion levels across different thermal environments (*p* = 0.010), and by thermal environments across production cycles (*p* = 0.001) (Table 3, Figure 4).

Birds exposed to severe heat stress (36 °C) exhibited the highest mean cloacal temperatures (Figure 4A). However, phytase supplementation at 500 FTU led to a reduction in rectal temperature, with phytase overdosing (1000, 1500, and 3000 FTU) further decreasing this parameter. Notably, at higher supplementation levels, cloacal temperatures of quails under severe heat stress (36 °C) approached those of quails maintained at 30 °C, indicating a potential thermoregulatory benefit. A similar trend was observed in quails kept under thermoneutral conditions (24 °C), where 500 FTU supplementation contributed to a decrease in rectal temperature, while higher phytase doses (1000 FTU and above) resulted in a significant reduction with no differences among them. No significant effect of phytase inclusion was observed for birds housed in the moderate heat stress environment (30 °C, *p* = 0.135) (Figure 4A).

At the beginning of the laying period (cycle 1), birds exhibited lower cloacal temperature values, which increased progressively throughout the evaluation period across all thermal environments (Figure 4B). No significant differences in cloacal temperature were observed between the birds exposed to moderate (30 °C) and severe heat stress (36 °C) at the beginning and end of the study. However, at the peak of laying (cycle 3), cloacal temperature values differed significantly across thermal environments (Figure 4B).

**Figure 5:**
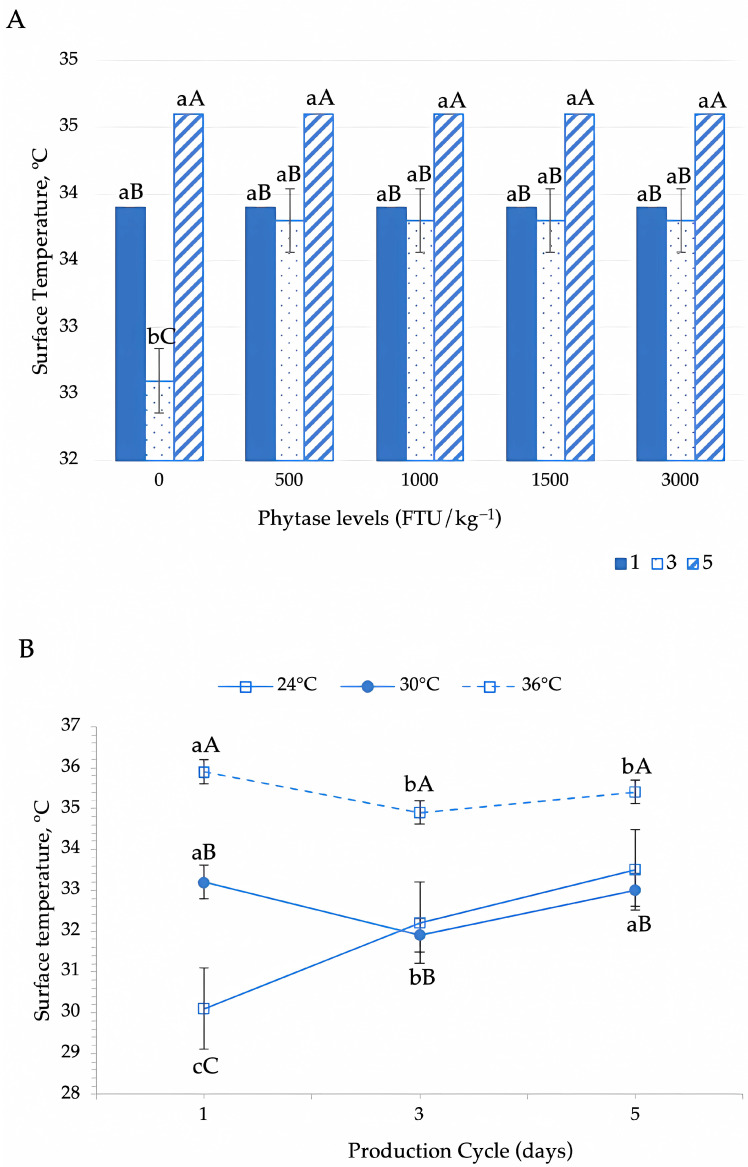
Dynamics of surface temperature in Japanese quails in response to phytase enzyme supplementation across production cycles (**A**) and different thermal environments during the production phase (**B**). In (**A**), different lowercase letters indicate differences between phytase levels in each evaluation cycle and different uppercase letters indicate differences between cycles within each phytase level according to Tukey’s test (*p* < 0.05). In (**B**), means followed by different lowercase letters (horizontal comparisons) and uppercase letters (vertical comparisons) differ significantly according to Tukey’s test (*p* < 0.05).

The surface temperature of the birds was significantly influenced by phytase supplementation across production cycles (*p* = 0.001) (Table 3). The lowest surface temperatures were observed at the beginning of laying (cycle 1) and during the peak laying of the birds (cycle 3), while the highest surface temperatures occurred in cycle 5 (Figure 5A). During cycle 3, characterized by the highest egg production, an increase in phytase concentration (500 FTU) led to a significant rise in the surface temperature. On the other hand, no significant effect of phytase supplementation on surface temperature was observed during cycle 1 (initial laying phase) and cycle 5 (declining laying phase) (Figure 5A).

The surface temperature of the birds was significantly influenced by environmental temperatures and across production cycles (*p* < 0.001) (Table 3, Figure 5B). At the beginning of laying (cycle 1), birds exposed to heat stress environments (30 °C and 36 °C) exhibited higher surface temperatures, whereas those in the thermoneutral environment (24 °C) had lower surface temperatures. During peak production (cycle 3), surface temperature increased in birds from the thermoneutral environment (24 °C), while birds exposed to heat stress conditions (30 °C and 36 °C) showed a reduction in the surface temperature. No significant difference in surface temperature was observed between birds in the thermoneutral (24 °C) and moderate heat stress (30 °C) at peak laying (cycle 3) (Figure 5B). In cycle 5, surface temperature increased in the thermoneutral and moderate heat stress environments. Birds in the severe heat stress environment (36 °C) exhibited the highest surface temperatures on average, whereas no significant differences were observed between birds in the thermoneutral and moderate heat stress environments (Figure 5B).

**Figure 6:**
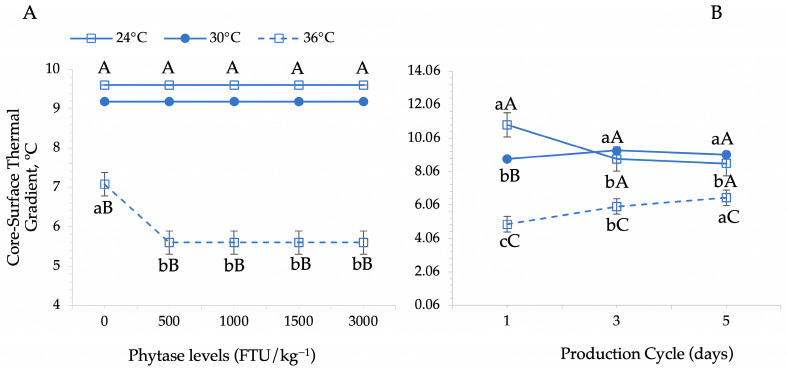
Dynamics of the core–surface thermal gradient in Japanese quails in response to phytase enzyme supplementation across thermal environments (**A**) and environmental temperature variations during production cycles (**B**). Means followed by different lowercase letters (horizontal comparisons) and uppercase letters (vertical comparisons) differ significantly according to Tukey’s test (*p* < 0.05).

**Figure 7:**
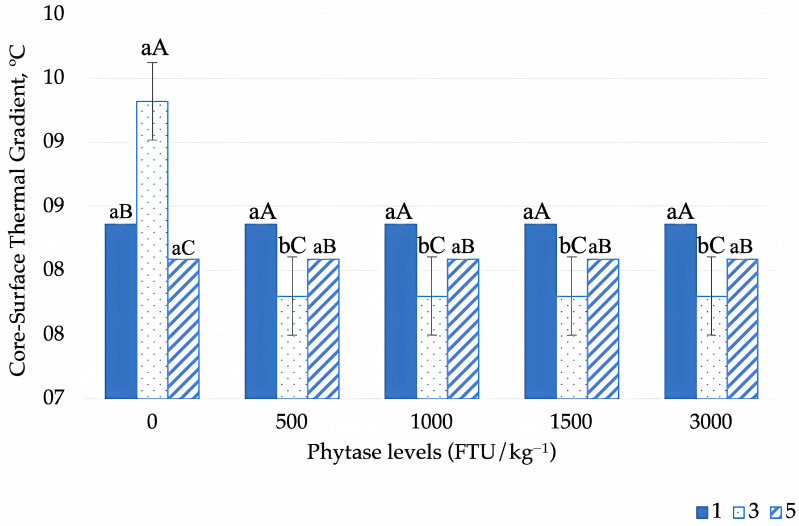
Dynamics of the core–surface thermal gradient in Japanese quails in response to phytase enzyme supplementation across production cycles. Different lowercase letters indicate differences between phytase levels in each evaluation cycle, and different uppercase letters indicate differences between cycles within each phytase level according to Tukey’s test (*p* < 0.05).

The core–surface thermal gradient was significantly influenced by phytase supplementation across different thermal environments (*p* = 0.002) and across production cycles (*p* = 0.001). In addition, core–surface thermal gradient was significantly affected by environmental temperature across the production cycles (*p* < 0.001) (Table 3, Figure 6 and Figure 7). Birds in the thermoneutral (24 °C) and moderate heat stress (30 °C) environments showed, on average, a higher core–surface thermal gradient, regardless of phytase levels. In contrast, birds exposed to severe heat stress exhibited a phytase-dependent reduction in this gradient (Figure 6A). Phytase supplementation at 500 FTU effectively reduced the core–surface thermal gradient in birds subjected to severe heat stress, but no further reductions were observed with higher phytase doses (1000 FTU and above) (Figure 6A).

The highest core–surface thermal gradient values were observed during the first evaluation cycle in birds housed in the thermoneutral environment (Figure 6B); however, these values gradually declined as the production cycles progressed. Conversely, birds in the heat stress environments exhibited a progressive increase in the core–surface thermal gradient over time. Despite the overall increase, the lowest values within these environments were consistently observed in the severe heat stress environment.

When analyzing the effect of phytase levels during the production cycles, we can observe that the highest values for the core–surface thermal gradient were verified during the first and last cycles compared to the laying peak (cycle 3) in birds that were fed the diet supplemented by the different phytase levels (500, 1000, 1500, and 3000 FTU). Birds that did not receive the phytase supplementation during the laying peak (cycle 3) had higher core–surface thermal gradient values on average (*p* < 0.05). The inclusion of 500 FTU phytase in the diet significantly reduced this gradient, though no further differences were observed with higher phytase doses (overdoses) (Figure 7).

**Figure 8:**
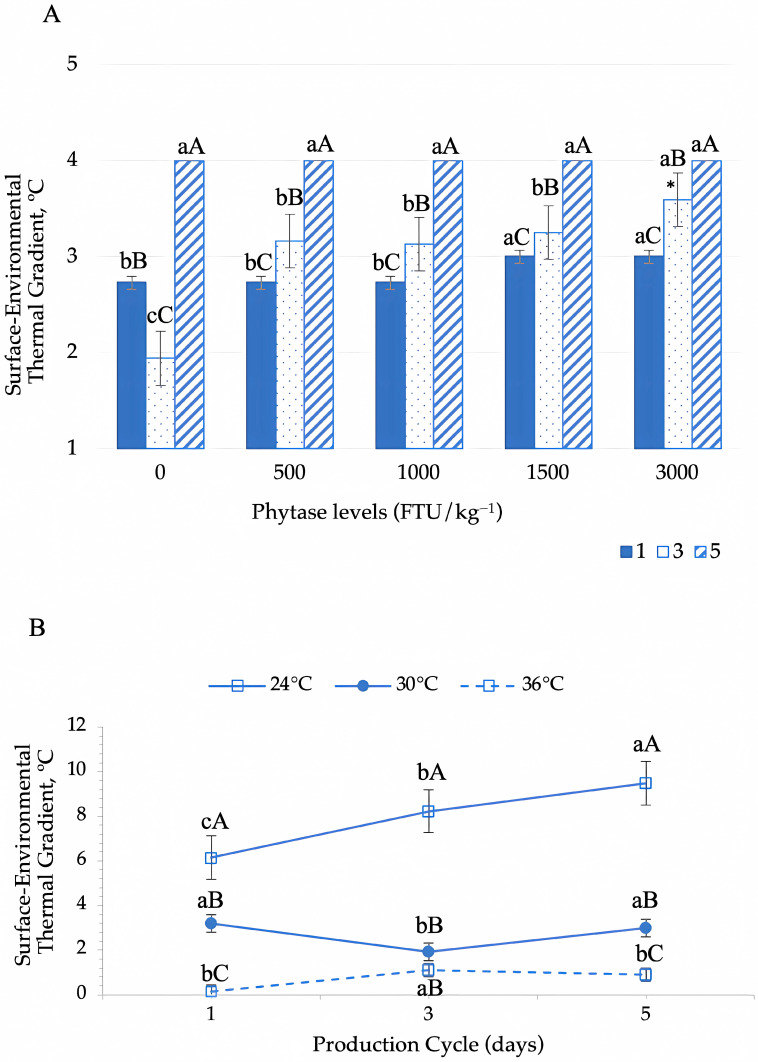
Dynamics of the surface–environment thermal gradient in Japanese quails in response to phytase enzyme supplementation during the production period (**A**) and across thermal environments (**B**). In (**A**), different lowercase letters indicate differences between phytase levels in each evaluation cycle and different uppercase letters indicate differences between cycles within each phytase level according to Tukey’s test (*p* < 0.05). In (**B**), means followed by different lowercase letters (horizontal comparisons) and uppercase letters (vertical comparisons) differ significantly according to Tukey’s test (*p* < 0.05). * Linear regression: surface–environment thermal gradient = 0.0004x + 2.4998; R^2^ = 0.62; *p* = 0.036.

The surface-to-environment thermal gradient was significantly influenced by phytase supplementation levels across production cycles (*p* < 0.001) (Figure 8A) and by environmental temperature variations during the evaluation period (*p* < 0.001) (Figure 8B). During the first production cycle, phytase inclusion at 1500 and 3000 FTU increased the surface-to-environment thermal gradient. At peak laying (cycle 3), birds that did not receive phytase supplementation exhibited lower values for this gradient. However, phytase overdosing (3000 FTU) led to a significant increase in this parameter (Figure 8A). In the final production cycle, the highest mean values for this gradient were observed; however, no significant effect of phytase supplementation was detected.

Quails housed in the thermoneutral environment exhibited a progressive increase in the surface-to-environment thermal gradient throughout the production cycles, maintaining higher values than those in the moderate and severe heat stress environments (Figure 8B). In contrast, birds in the heat stress environments showed opposing trends during the first evaluation cycle. However, no significant differences were observed between these groups at peak laying. By the final production cycle, quails in the moderate stress environment exhibited the highest values for this gradient, whereas those in the severe heat stress environment showed a decline in surface–environment thermal gradient values.

## 4. Discussion

### 4.1. Thermoregulatory Response

Under thermoneutral (24 °C) and moderate heat stress (30 °C) conditions, phytase supplementation did not significantly affect convective heat exchange, with quails maintaining a constant mean value of 147.1 W/m^2^ and 104.7 W/m^2^, respectively, regardless of enzyme inclusion. This suggests that, at temperatures ranging from 24 °C to 30 °C, phytase supplementation is not essential for regulating convective heat loss, even though birds in a thermoneutral environment rely more heavily on this mechanism.

In severe heat stress environments (36 °C), convective heat exchange was influenced by phytase levels in the diet. The inclusion of 500 FTU in the diet increased convective heat loss in birds exposed to this environment. However, higher phytase overdoses (1500 and 3000 FTU) enhanced the efficiency of convective heat transfer. These findings are consistent with those reported by Matos Júnior et al., who observed that convective heat loss was significantly greater in thermoneutral (24 °C) environments compared to 32 °C conditions also in Japanese quails. Our results indicate that the estimated optimal dose of phytase under severe heat stress (36 °C) was 2221 FTU/kg (Y = 62.99 + 0.0201·X − 4.533 × 10^−6^·X^2^; R^2^ = 0.91; *p* = 0.014).

Phytase supplementation also influenced radiative heat exchange. Under thermoneutral and moderate heat stress conditions, the inclusion of phytase did not significantly affect this response. However, under severe heat stress conditions, phytase overdose increased the reliance on radiative heat exchange compared to quails fed control diets (both positive and negative controls). According to the regression model, the suggested dose for this environment was approximately 2500 FTU/kg, with no additional benefit observed at higher levels. Like convection, radiation is a physical mechanism of sensible heat dissipation, occurring when internally generated heat reaches peripheral body regions, especially those lacking feather coverage, and is emitted as infrared radiation.

These findings suggest that phytase supplementation plays a crucial role in modulating heat dissipation mechanisms in laying quails exposed to varying thermal environments and that it may serve as a valuable nutritional strategy for modern poultry farming.

The action of phytase in breaking down phytic acid not only releases important minerals such as phosphorus but also liberates other nutrients chelated to this molecule, thereby enhancing the nutritional value of the diet. This improved nutrient availability is particularly beneficial under thermally challenging conditions, where birds often reduce feed intake as a thermoregulatory strategy [2,5,15].

Our study also showed that birds housed under thermoneutral conditions exhibited a greater dependence on convective and radiative heat dissipation compared to those under severe heat stress. This increased reliance on sensible heat exchange mechanisms under comfortable temperatures suggests more effective thermal regulation, likely supported by digestion and metabolism functioning optimally in the absence of thermal stress. In such conditions, birds are not required to activate more energetically demanding mechanisms to maintain thermal homeostasis. A fundamental physiological adaptation in homeothermic animals under heat stress is the reduction in feed intake, aimed at minimizing metabolic heat production. Although we did not observe a significant effect of phytase inclusion under thermoneutral and moderate heat stress conditions, the lower heat generated by physiological and digestive processes, combined with phytase supplementation, likely facilitated peripheral blood perfusion and heat dissipation from the core to the surface [23].

At the beginning of the laying phase, under thermoneutral conditions (24 °C), birds relied less on convective and radiative heat exchange compared to later production cycles. Initially, quails housed in the thermoneutral environment depended less on radiative heat dissipation than those exposed to high temperatures. However, as birds reached peak laying, radiative heat dissipation declined in heat-stressed environments, while it increased in thermoneutral conditions. After peak laying, birds in cooler environments continued to utilize radiative heat exchange more efficiently than those in hotter conditions. Interestingly, during cycle 5 (declining phase of egg production), radiative heat dissipation increased in birds exposed to thermal stress (30 °C and 36 °C), possibly as a compensatory response.

Our results also highlight the effectiveness of infrared thermographic imaging technology in evaluating thermoregulatory responses, suggesting that this tool can be employed in production systems as a practical and non-invasive method for detecting thermal discomfort in birds. Since blood circulation influences heat dissipation, thermographic imaging enables the detection of changes in peripheral blood flow, where increased circulation enhances radiative emissivity, which can be quantified through thermal image analysis [24].

One unaccounted factor in this study is the role of latent heat transfer via respiratory evaporation (panting), which becomes critical when convection and radiation are insufficient under extreme heat stress conditions [4,25]. However, in poultry production systems, maintaining optimal environmental conditions is essential to support heat dissipation through sensible mechanisms (convection and radiation). Prolonged reliance on latent heat loss through panting leads to physiological stress, ultimately impairing bird welfare and production efficiency [26].

### 4.2. Physiological Response

Among the various physiological parameters, cloacal temperature is widely recognized as a reliable indicator of internal body temperature, providing insight into an animal’s thermal comfort or heat stress status. Several factors, including age, weight, sex, activity level, feed intake, and thermal environment, influence the cloacal temperature in birds [12]. Our findings indicate that birds housed under severe heat stress and not supplemented with phytase exhibited higher cloacal temperatures on average. However, as phytase supplementation increased, particularly at overdose levels (1000, 1500, and 3000 FTU), cloacal temperature significantly decreased. A similar trend was observed in birds housed in the thermoneutral conditions, where phytase supplementation effectively reduced cloacal temperature, especially at higher inclusion levels. Although cloacal temperature varied significantly across thermal environments, quails remained within the normal physiological range of 40–42 °C [27,28,29]. These results suggest that phytase supplementation plays a role in thermoregulation, potentially contributing to improved heat dissipation and metabolic efficiency in laying quails under different environmental conditions.

The increase in air temperature from 24 °C to 36 °C is likely responsible for the elevated surface temperatures observed in birds subjected to severe heat stress. Birds housed in high-temperature environments often experience significant plumage loss in the dorsal region (personal observations), which can exacerbate heat dissipation. Under heat stress conditions, birds tend to redirect blood flow to peripheral tissues, facilitating heat transfer from the body’s core to the featherless surface regions. This physiological adaptation enhances sensible heat dissipation through convection and radiation, playing a crucial role in maintaining thermal balance [30,31]. Beyond cloacal and surface temperatures, the core-to-surface and surface-to-environment thermal gradients serve as valuable physiological indicators for assessing thermal comfort or heat stress in birds. These gradients provide insights into heat exchange efficiency between the animal and the environment, offering a more comprehensive evaluation of thermoregulatory responses [12].

The highest core–surface thermal gradient values were observed in birds housed in thermoneutral and heat stress environments, regardless of phytase inclusion levels. However, phytase supplementation effectively reduced this gradient in birds subjected to severe heat stress (36 °C). A lower core–surface thermal gradient indicates a greater similarity between internal body temperature and surface temperature, suggesting that birds require more efficient thermoregulatory responses to facilitate heat dissipation. Under these conditions, birds must enhance thermolysis mechanisms, supplementing sensible heat loss through convection and radiation with additional physiological adaptations, such as increased peripheral blood flow or evaporative cooling.

The surface temperature of quail is directly correlated with the ambient temperature, as efficient heat dissipation requires a temperature gradient between the bird’s body surface and its environment. Heat naturally flows from warmer to colder areas, facilitating thermal regulation [8]. The surface-to-environment thermal gradient quantifies the bird’s heat dissipation capacity, reflecting the difference between body surface temperature and ambient temperature. Our findings indicate that during the initial laying phase, birds supplemented with phytase overdoses (1500 and 3000 FTU) exhibited a higher thermal gradient, suggesting enhanced thermolysis efficiency compared to other groups.

During peak laying, birds experience increased metabolic heat production due to the high physiological demand for egg production. Our results indicate that phytase supplementation at 3000 FTU led to a linear increase in the surface-to-environment thermal gradient, suggesting enhanced heat dissipation efficiency. Additionally, birds housed in the thermoneutral environment (24 °C) exhibited a progressive increase in thermal exchange efficiency over the production cycles, as evidenced by the gradual rise in the surface-to-environment thermal gradient. This finding highlights the adaptive thermoregulatory advantage provided by a stable thermal environment, allowing birds to maintain efficient heat dissipation mechanisms throughout the laying period.

The efficiency of heat exchange through sensible mechanisms is directly proportional to the temperature differential between the bird’s surface and the surrounding environment. As observed in this study, heat flux via sensible mechanisms relies on a favorable thermal gradient, as a narrower temperature difference reduces the effectiveness of heat dissipation [32]. At an ambient temperature of approximately 21 °C, birds can dissipate up to 75% of their metabolic heat through sensible pathways. However, as the ambient temperature approaches the bird’s surface temperature, the sensible mechanisms become less effective, shifting thermoregulation reliance toward evaporative mechanisms, particularly respiratory evaporation [33,34]. This transition underscores the importance of maintaining a sufficient thermal gradient to optimize heat dissipation and prevent thermal stress in poultry production systems.

## 5. Conclusions

The thermal environment critically influences the physiological responses and welfare of Japanese quails during the production cycle. Our results demonstrated that dietary supplementation with phytase above 1500 FTU/kg^−1^ in a severe heat stress environment (36 °C) effectively contributes to heat dissipation through convective (2221 FTU) and radiative (2500 FTU) mechanisms, aiding in the thermoregulation of Japanese quails during the egg production phase.

Additionally, phytase overdosing significantly reduced the cloacal temperature and optimized core-to-surface and surface-to-environment thermal gradients, indicating improved thermal homeostasis. These findings suggest that nutritional interventions with exogenous enzymes can mitigate the negative impact of heat stress, thereby promoting greater physiological resilience in quails exposed to elevated ambient temperatures.

Overall, the strategic inclusion of phytase overdoses represents a viable nutritional tool to enhance heat tolerance and welfare in quails, mitigating the adverse effects of heat stress and supporting the optimal performance during the production phase.

## Figures and Tables

**Table 1:** Composition and nutritional profile of experimental diets formulated for laying Japanese quails.

Ingredients (kg)	Phytase Levels (FTU/kg^−1^)				
	0	500	1000	1500	3000
Corn grain, 7.88%	59.72	59.72	59.72	59.72	59.72
Soybean bran, 45%	30.51	30.51	30.51	30.51	30.51
Soybean oil	0.667	0.667	0.667	0.667	0.667
Calcitic limestone	7.437	7.437	7.437	7.437	7.437
Dicalcium phosphate, 18.5%	0.400	0.400	0.400	0.400	0.400
Common salt	0.345	0.345	0.345	0.345	0.345
DL-methionine	0.398	0.398	0.398	0.398	0.398
L-lysine HCl	0.265	0.265	0.265	0.265	0.265
L-threonine	0.035	0.035	0.035	0.035	0.035
Choline chloride, 60%	0.070	0.070	0.070	0.070	0.070
Mineral premix ^a^	0.050	0.050	0.050	0.050	0.050
Vitamin premix ^b^	0.025	0.025	0.025	0.025	0.025
Coccidiostat (Coxistac) ^c^	0.010	0.010	0.010	0.010	0.010
Phytase ^d^	0.000	0.010	0.020	0.030	0.060
Inert	0.060	0.050	0.040	0.030	0.000
Total	100	100	100	100	100
Chemical Composition					
Crude protein (%)	19	19	19	19	19
Metabolizable energy (kcal kg^−1^)	2800	2800	2800	2800	2800
Methionine + digestible cysteine (%)	0.908	0.908	0.908	0.908	0.908
Digestible lysine (%)	1.107	1.107	1.107	1.107	1.107
Digestible threonine (%)	0.675	0.675	0.675	0.675	0.675
Digestible valine (%)	0.798	0.798	0.798	0.798	0.798
Tryptophan (%)	0.207	0.207	0.207	0.207	0.207
Calcium (%)	2.993	2.993	2.993	2.993	2.993
Available phosphorus (%)	0.177	0.177	0.177	0.177	0.177
Sodium (%)	0.155	0.155	0.155	0.155	0.155
Chlorine (%)	0.319	0.319	0.319	0.319	0.319
Potassium (%)	0.732	0.732	0.732	0.732	0.732
Mongin number	164.6	164.6	164.6	164.6	164.6

^a^ Mineral premix (per kg): 10 mg of Cu, 50 mg of Fe, 80 mg of Mn, 1.2 g of I. ^b^ Vitamin premix (per kg): 8.000 IU of vitamin A, 2.000 IU of vitamin D3, 15 mg of vitamin E, 2 mg of vitamin K, 3 mg of vitamin B1, 4 mg of vitamin B2, 2 mg of vitamin B6, 10 mg of vitamin B12, 60 mg of biotin, 15 mg of pantothenic acid, 30 g of niacin, 7 mg of folic acid, 4 g of selenium. ^c^ Salinomycin 12%, Phibro. ^d^ Phytase Quantum Blue, AB Vista^®^, Marlborough, UK.

**Table 2:** Heat transfer by convection and radiation in Japanese quails raised in different thermal environments with phytase supplementation during the production phase.

Phytase Levels, FTU/kg^−1^	Heat Transfer by Convection, W/m^2^	Heat Transfer by Radiation, W/m^2^
0	104.7	39.06
500	108.6	40.86
1000	108.0	41.63
1500	109.7	39.96
3000	113.1	42.13
Environments, °C		
24	148.76	46.98
30	103.6	37.78
36	76.02	37.44
Production cycle		
Laying begins (1)	106.13	39.48
Posture peak (3)	107.06	39.26
Posture post-peak (5)	113.36	43.44
Sources of variation	*p*-value ^a^	
Phytase levels (P)	0.010 *	0.038 *
Environments (E)	<0.001 **	<0.001 **
Production cycle (C)	<0.001 **	0.001 *
P × E	0.001 *	0.029 *
P × C	0.139	0.171
E × C	0.001 *	<0.001 **
P × E × C	0.759	0.070

^a^ Statistical significance is defined as *p* < 0.05; values above this threshold are considered non-significant. * *p* < 0.05; ** *p* < 0.001.

**Table 3:** Physiological thermoregulation parameters of laying Japanese quails (*Coturnix japonica*) raised in different thermal environments under phytase enzyme supplementation.

Phytase Level, FTU/kg^−1^	Cloacal Temperature, °C	Surface Temperature, °C	Core– Surface Thermal Gradient	Surface– Environment Thermal Gradient
0	38.65	33.06	5.59	3.24
500	41.37	33.30	8.07	3.29
1000	41.43	33.53	7.90	3.55
1500	41.33	33.30	8.03	3.28
3000	41.36	33.50	7.86	3.50
Environments, °C				
24	41.18	31.93	9.41	7.93
30	41.34	32.70	9.06	2.70
36	41.76	37.40	5.78	1.40
Production cycle				
Laying begins (1)	41.05	33.08	8.18	3.16
Posture peak (3)	41.26	33.00	8.05	3.01
Posture post-peak (5)	41.90	33.94	7.96	3.95
Sources of variation		*p*-value ^a^		
Phytase levels (P)	0.016 *	0.048 *	0.001 *	0.358
Environments (E)	<0.001 **	<0.001 **	<0.001 **	<0.001 **
Production cycle (C)	<0.001 **	<0.001 **	0.485	<0.001 **
P × E	0.010 *	0.457	0.002 *	0.974
P × C	0.325	0.001 *	0.001 *	<0.001 **
E × C	0.001 *	<0.001 **	<0.001 **	<0.001 **
P × E × C	0.413	0.079	0.192	0.351

^a^ Statistical significance is defined as *p* < 0.05; values above this threshold are considered non-significant. * *p* < 0.05; ** *p* < 0.001.

## Data Availability

Data are available from the corresponding author upon request.
